# Restoration of Regenerative Osteoblastogenesis in Aged Mice: Modulation of TNF

**DOI:** 10.1359/jbmr.090708

**Published:** 2009-07-06

**Authors:** Elizabeth C Wahl, James Aronson, Lichu Liu, John L Fowlkes, Kathryn M Thrailkill, Robert C Bunn, Robert A Skinner, Mike J Miller, Gael E Cockrell, Lindsey M Clark, Yang Ou, Carlos M Isales, Thomas M Badger, Martin J Ronis, John Sims, Charles K Lumpkin

**Affiliations:** 1 Arkansas Children's Hospital Research Institute Little Rock, AR, USA; 2 Department of Orthopedics, College of Medicine, University of Arkansas for Medical Sciences Augusta, GA, USA; 3 Department of Pediatrics, College of Medicine, University of Arkansas for Medical Sciences Augusta, GA, USA; 4 Medical College of Georgia Augusta, GA, USA; 5 Department of Physiology/Biophysics, College of Medicine, University of Arkansas for Medical Sciences Seattle, WA, USA; 6 Department of Pharmacology/Toxicology, College of Medicine, University of Arkansas for Medical Sciences Seattle, WA, USA; 7 Amgen Seattle, WA, USA

**Keywords:** bone repair, aging, TNF, cytokine

## Abstract

Skeletal changes accompanying aging are associated with both increased risk of fractures and impaired fracture healing, which, in turn, is due to compromised bone regeneration potential. These changes are associated with increased serum levels of selected proinflammatory cytokines, e.g., tumor necrosis factor α (TNF-α). We have used a unique model of bone regeneration to demonstrate (1) that aged-related deficits in direct bone formation can be restored to young mice by treatment with TNF blockers and (2) that the cyclin-dependent kinase inhibitor p21 is a candidate for mediation of the osteoinhibitory effects of TNF. It has been hypothesized recently that TNF antagonists may represent novel anabolic agents, and we believe that the data presented here represent a successful test of this hypothesis. © 2010 American Society for Bone and Mineral Research

## Introduction

The National Osteoporosis Foundation has estimated that osteoporosis is a major public health threat for over 50% of the population older than 50 years of age. Even patients in this population who are taking potent pharmacologic substances to lower their fracture risk remain at risk for fracture.(1) In fact, clinical and experimental evidence suggests that the entire aged human population is at risk for impaired bone repair/regeneration.(1)

In humans, rats, and mice, fracture healing slows with increasing age.(2–4) Fractures of the femur heal more quickly in younger than in older children, whereas mandibular fractures heal faster in humans younger than than 18 years of age than in those older than 18.(3) Aging in adults is associated with greater time to union for tibial and humeral fractures, as well as for floating knee injuries.(3) In adults older than 50 years of age, increasing age correlates with reduction of the percent of unions at 6 months after injury. A recent study of patients 35 to 92 years of age demonstrated that short-term morbidity was common to all fractures, with varying degrees of prolonged morbidity often extending to at least a year postfracture.(3) First fractures also significantly increase the probability of subsequent fractures. It also has been noted that age-related mortality/morbidity owing to fractures is higher in males than in females.(4)

Most fractures heal by a combination of endochondral and direct (intramembranous, appositional) bone formation. In fractures, direct bone formation results in the hard callus that is responsible for the initial bridging and stabilization of the fracture. In order to focus on direct bone formation in an aged context, we have developed a unique murine bone-regeneration protocol that falls in the category of distraction osteogenesis (DO). This model is a direct analogue of the clinical DO (limb-lengthening) protocol. DO can be considered a variant of fracture healing that significantly expands the volume of new bone formed and challenges the proliferative capacity of the animal. Direct bone formation in fracture and DO shares many common features at the cellular and molecular levels.(5) In fact, fatigue, stress, and minimally displaced fractures all appear to heal by direct bone formation.(6,7) Taken together, the results of these studies may explain the close correspondence between DO and fracture healing demonstrated in models of chronic ethanol exposure, lead exposure, diabetes, and aging.(8–11) It also should be noted that DO is used to treat delayed union, nonunion, and infected fractures.(12,13) Therefore, we postulate that deficits in fracture healing involving direct bone formation in the clinic can be modeled by DO in the laboratory.

Clinical experience with the DO technique has revealed an age-related decrement in new bone density and in healing index.(14–16) These measures can be translated into an age-related decrease in the rate of bone formation. In fact, in one study, patient age was the most important factor in determining new bone density.(15) It has been demonstrated that the histologic pattern of bone formation by DO in dogs, rabbits, rats, and mice appears similar to that in humans.(17,18) This suggests that rodent models of DO will be useful as analogues to the human DO process and in investigating alterations in direct bone formation and repair during aging. In fact, recent work has demonstrated age-related deficits in DO in both rat and mouse models at 12 months of age or greater.(8,19)

The DO process can be divided into two major phases. First is the distraction phase, where the edges of a tibial fracture are stretched apart, and where processes of direct bone formation predominate in the slowly widening gap. This is followed by the consolidation phase, beginning after the stretching stops, where bone bridging (continuous osteoid/bone “bridge” from proximal to distal cortices) and the osteoclast-mediated processes of bone resorption and remodeling are initiated at each host bone surface to re-form the medullary canal and to replace woven with lamellar bone. Actually, the presence of osteoclasts (TRAP+ multinucleated cells) is first detected when the expanding DO gap is almost bridged to begin re-formation of the medullary canal.

In the clinical population TNF, interleukin 6 (IL-6), and IL-1 have been consistently demonstrated to be increased in serum in the aged population. Aging has been characterized as “inflamm-aging,” and a new discipline named *osteoimmunology* has arisen in response to the high levels of these and other cytokines in the aging population.(20,21)

TNF is produced primarily as a product of the monocyte-macrophage cell lineage and is responsible for proliferation, inhibition, differentiation, and activation of a variety of cell types.(22) It also has been shown to be synthesized by bone marrow stromal cells, T cells, and osteoblasts. TNF commonly refers to two soluble proteins, TNF-α and TNF-β (lymphotoxin α), which have a high degree of sequence homology and share receptors. TNF-α exists in soluble and transmembrane forms (tmTNF). TNF interacts with two receptors, TNF-R1 and -R2. Most of TNF's effects have been attributed to TNF-R1. Naturally occurring TNF antagonists can be found in soluble forms, which are derived from the extracellular domains of TNF-R1 (sTNFR1) or -R2. These soluble receptors and/or commercially produced antibodies to TNF also may bind to the tmTNF form, resulting in what is termed *reverse signaling* (i.e., sTNFR1 binds tmTNF, which initiates signal-transduction pathways). In this article, sTNFR1 will refer specifically to two molecules of the extracellular domain of the human TNF-R1 linked to a molecule of polyethylene glycol (Pegsunercept),(19) whereas sTNFR2 will refer to two molecules of the extracellular domain of the human TNF-R2 linked to the Fc portion of human IgG1 (Etanercept/ Enbrel).

Previous studies have demonstrated the ability of TNF to inhibit multiple osteoblast functions in vitro as well as fracture repair in vivo.(22–24) The signal-transduction pathways activated by TNF binding to its receptors have been studied extensively in several systems.(25) In regard to TNF effects on osteoblastogenesis in vitro, recent work using fetal rat calvarial cells and a murine calvarial osteoblastic cell line has demonstrated that TNF (1) is a potent inhibitor of osteoblast differentiation from precursor cells, (2) acts distal to insulin-like growth factor I (IGF-I) and bone morphogenetic proteins (BMPs), (3) inhibits the expression of RUNX2 and Osterix (osteoblast-associated transcription factors) through MEK1 kinases, (4) suppresses vitamin D–stimulated transcription owing to activation of transcription factor NFκB, and (5) actions are mediated by TNFR1.(26–28) Though high levels of TNF are known to inhibit direct bone formation in culture and in vivo, nevertheless, low doses can enhance osteoblast proliferation in culture, and impaired bone formation has been demonstrated in TNFR1/R2 double-knockout mice.(25,26) This suggests that a homeostatic level of TNF signaling is required for optimal bone formation but that unregulated or excessive expression results in pathology.

Further, in the paradigms named *replicative, premature*, and *cytokine-driven senescence*, TNF signaling through TNFR1 has been shown to induce p53, which, in turn, activates a cyclin-dependent kinase inhibitor, i.e., p21 (Sdi1, WAF1, Cip1, Cdkn1a), and a senescence marker in various cell types.(22,31,32) Recently, our colleagues have demonstrated that the effects of long-term EtOH exposure on rat stromal osteoblasts in vitro include induction of p53, p21, and a senescent marker.(33) These findings, coupled with our published work demonstrating that chronic ethanol exposure inhibits direct bone formation during distraction through the TNF signaling axis, suggested to us that induction of p21 activity in aged mice undergoing DO may provide one direct molecular and functional link between aging, premature senescence, chronic ethanol exposure, and osteoinhibition. The availability of p21 knockout (KO) mice and the fact that their skeletal development is normal presented the opportunity for us to test for hypothetical p21 mediation of TNF effects in the DO model (JAX on line, #003263).

In order to test the hypothesis that the elevated levels of TNF associated with aged animals mediate the age-related inhibition of direct bone formation characteristic of fracture repair and DO, we have applied the DO bone-regeneration model to mice of varying ages. Previous studies using this DO model have demonstrated that aging is associated with elevated serum TNF levels, inhibition of direct bone formation, and inhibition of proliferation markers in osteoblast precursors.(8,19) Further, treatment of young mice with recombinant mouse tumor necrosis factor (rmTNF) both inhibits bone formation and decreases proliferation markers during DO.(34,35) These results, coupled with the crucial dependence of DO on sustained cellular proliferation, suggest that aging in these models may be associated with antiproliferative factors such as the known TNF-inducible genes *p53* and *p21*.(27,31) These results also suggest that direct bone formation in the aged mouse DO model is analogous to the rat and human and therefore can be used to further study the effects TNF signal modulation in aged mice.

Here we test the hypothesis that the elevated levels of TNF associated with aged animals mediate the age-related inhibition of bone formation demonstrated in fracture repair and DO. We employed the mouse DO model to (1) test the effects of TNF blockers on direct bone formation in aged animals and (2) test for a potential role for p21 in the exogenous rmTNF-associated osteoinhibition.

## Materials and Methods

### Animals

Virus-free adult male C57BL/6 mice were purchased from Jackson Labs (Bar Harbor, ME). They were housed in individual cages in temperature- (22°C) and humidity-controlled (50%) rooms having a 12 hours light per 12-hour dark cycle. All mice were handled by animal care personnel for 5 to 7 days prior to surgery. In all studies, the mice were assigned to respective experimental groups with mean body weights equal to that of the control group (±4 g). All research protocols were approved by the Institutional Animal Care and Use Committee (IACUC) of the University of Arkansas for Medical Sciences.

### Distraction protocol

Following acclimation and under Nembutal anesthesia, each mouse underwent placement of an external fixator and osteotomy to the left tibia.(19,35,36) Four 27-gauge, 1.25-in needles were manually drilled through the tibia (two proximally, two distally). The titanium external fixator then was secured to the pins. A small incision was made in the skin distal to the tibial crest, and the soft tissue was carefully retracted to visualize the bone. A single hole was manually drilled through both cortices of the mid-diaphysis, and surgical scissors were used to fracture the cortex on either side of the hole. The fibula was fractured by direct lateral pressure. The periosteum and dermal tissues were closed with a single suture. Finally, buprenex (0.2 mg/kg) was given by intramuscular injection after surgery for analgesia. Distraction began 3 days after surgery (3-day latency) at a rate of 0.075 mm b.i.d. (0.15 mm/day) and continued for 14 days. After the distraction period, the mice were sacrificed, and the distracted tibiae were harvested for radiographic and histologic analyses. Trunk blood was collected for serum analyses.

### Radiographic/histologic analyses

After 48 hours of fixation in 10% neutral buffered formalin, the left tibiae were removed from the fixators for high-resolution single-beam radiography and subsequent histologic processing. For initial radiography, a Xerox Micro50 closed-system radiography unit (Xerox, Pasadena, CA, USA) was used at 40 kV (3 mA) for 20 seconds using Kodak X-OMAT film. For quantification, the radiographs were videorecorded under low-power (2× objective) microscopic magnification, and the area and density of mineralized new bone in the distraction gaps were evaluated by NIH Image Analysis 1.62 software/Image J software 1.30 (rsb.info.nih.gov/ij/). The measured distraction gap was outlined from the outside corners of the two proximal and two distal cortices, forming a quadrilateral region of interest. The mineralized new bone area in the gap was determined by outlining the regions with radiodensity equivalent to or greater than the adjacent medullary bone. The percentage of new mineralized bone area within the distraction gap (percent new bone) was calculated by dividing mineralized bone area by total gap area. Therefore, the percent new bone, as measured by the radiograph analysis, is an estimate of new “mineralized” bone in the entire gap.(35,37)

After radiography, the distracted tibiae were decalcified in 5% formic acid, dehydrated, and embedded in paraffin. Experience in our laboratory has demonstrated that this achieves good morphology in murine orthopedic tissues and does not appear to significantly impair immunologic detection of many epitopes.(38,39) Longitudinal sections 5 to 7 µm long were cut on a microtome (Leitz 1512, Wetzlar, Germany) for hematoxylin and eosin (H&E) staining. Sections were selected to represent a central or near-central gap location. As detailed earlier, a quadrilateral region of interest was outlined and recorded. Both the proximal and distal endocortical (measured from the inside corners of the cortices) and the intracortical (cortical wall included) new bone matrices were outlined together from the outside edges of the cortices, and the area was recorded as new gap bone formation. The percentage of new gap bone area within the DO gap (percent new bone) was calculated as earlier. The percent new bone as measured by the histologic analysis is an estimate of new gap bone formation, which would include nonmineralized osteoid columns, embedded new sinusoids, and maturing mineralized bone columns.(35) We note that the “fibrous interzone” that lies between the proximal and distal new bone formation fronts (i.e., the nonnew bone gap area) is comprised of fibroblastic cells overlaying parallel collagen bundles that are oriented along the distraction vector.

To be included in both radiographic and histologic analyses, the DO samples had to (1) be well aligned, (2) have no broken pin sites, (3) have no bone chips within the DO gap and the animals had to (4) have an intact ankle and (5) have had no significant weight loss or health problems during the distraction period.

### Experimental design

*Study 1:* To study the effects of a TNF antagonist on direct bone formation, 24 (22-month-old) and 10 (3-month-old) male C57BL/6 mice underwent the DO protocol. After the 3-day latency period, the young and half the aged mice received a subcutaneous injection of vehicle (phosphate-buffered saline, pH 7.4), whereas the remaining half of the aged mice received a subcutaneous injection of sTNFR1 (8.0 mg/kg) every other day for the 14-day distraction period. In a previous study, using the same protocols, *n* = 14 (9-month-old) male C57BL/6 mice received either vehicle or sTNFR1.

*Study 2:* A study comparing the effects of sTNFR1 and sTNFR2 (Etanercept/Enbrel) on direct bone formation in 21-month-old mice was performed. Thirty male C57BL/6 mice underwent the DO protocol and were divided into three equal groups: vehicle, sTNFR1-treated, and sTNFR2-treated. After the 3-day latency period, the mice received subcutaneous injections of either vehicle (PBS, pH 7.4), sTNFR1 (8.0 mg/kg), or sTNFR2 (8.0 mg/kg) every other day for the 14-day distraction period.

*Study 3:* A study comparing the effects of rmTNF on 3-month-old C57BL/6 versus *p21* KO (JAX #003263) mice was performed. Twenty-four male C57BL/6 and 24 male *p21* KO mice underwent the DO protocol. At the time of surgery, an Alzet pump (Model 1002) was inserted subcutaneously on the back of each mouse. The mice in the control groups (*n* = 12 each) received vehicle (PBS, pH 7.4) via Alzet, and those in the TNF groups (*n* = 12 each) received 10 µg/kg per day of rmTNF via Alzet pump (R&D Systems, Cat. No. 410-MT). Distraction began 3 days after surgery and continued for 14 days.

### Serum analyses

Serum samples were run using the Luminex xMAP technology in the Pediatric Endocrinology Core Facility. The Lincoplex mouse adipokine panels were used for TNF, IL-6, and insulin. Serum R&D DuoSet ELISA kits were used for DKK-1 and IGF-BP-2, 3, and 6. Serum R&D ELISA kits were used for human and mouse sTNFR1 and sTNFR2. Serum IGF levels were measured by IDS rat/mouse ELISA kits. Serum mouse osteocalcin levels were measured by a single-plex ELISA (Millipore).

### In vitro analyses

Primary calvaria cells were isolated from new born mice (*p21* KO or wild type). Calvaria were dissected and subjected to four sequential digestions in Earle's MEM containing 200 U/mL collagenase II at 37°C. Cells in fractions 3 and 4 were collected and combined. These calvarial cells were cultured at 3 to 5 × 10^5^ cells per T-75 cm^2^ flask in Earle's MEM containing 10% FBS. After 6 days, cells were trypsinized and subcultured in a 6-well plate at a density of 2 × 10^5^ cells per well. The following day, the medium was switched to serum-free medium containing 0.2% BSA for 16 hours. After the 16-hour serum starvation period, TNF-α (25 ng/mL) or vehicle solution was added to the medium. Twenty-four hours later, cells were lysed in RIPA buffer (50 mM Tris-HCl, pH 7.5, 4.5 mM NaCl, 0.25% sodium deoxycholate, and 1% NP-40) containing protease inhibitors. Proteins then were separated by 12% SDS-PAGE and electrotransferred to a PVDF membrane. The membrane was probed with rabbit anti-p21 antibody (C-19, Santa Cruz Biotechnology, Inc., CA, USA). Blots were developed using chemiluminescence according to the manufacture's instructions (Bio-Rad Immun Star Chemiluminescence Kit, Bio-Rad Laboratories, Hercules, CA, USA). Quantification of blots was performed using a VersaDoc imaging system (Bio-Rad Laboratories).

### Statistical analyses

For statistical analysis, differences between group means were determined by the Student's *t* test or by one-way ANOVA. Also, the Student-Newman-Keuls method was used for the pairwise multiple comparisons, and the *p* values agreed with the ANOVA values. All data are reported as mean ± standard error of the mean (SEM). Differences were considered significant when *P* < .05.

## Results

### Study 1: The effects of a TNF antagonist on direct bone formation in 22-month-old male C57BL/6 mice compared with 3-month-old mice

**Fig. 1:**
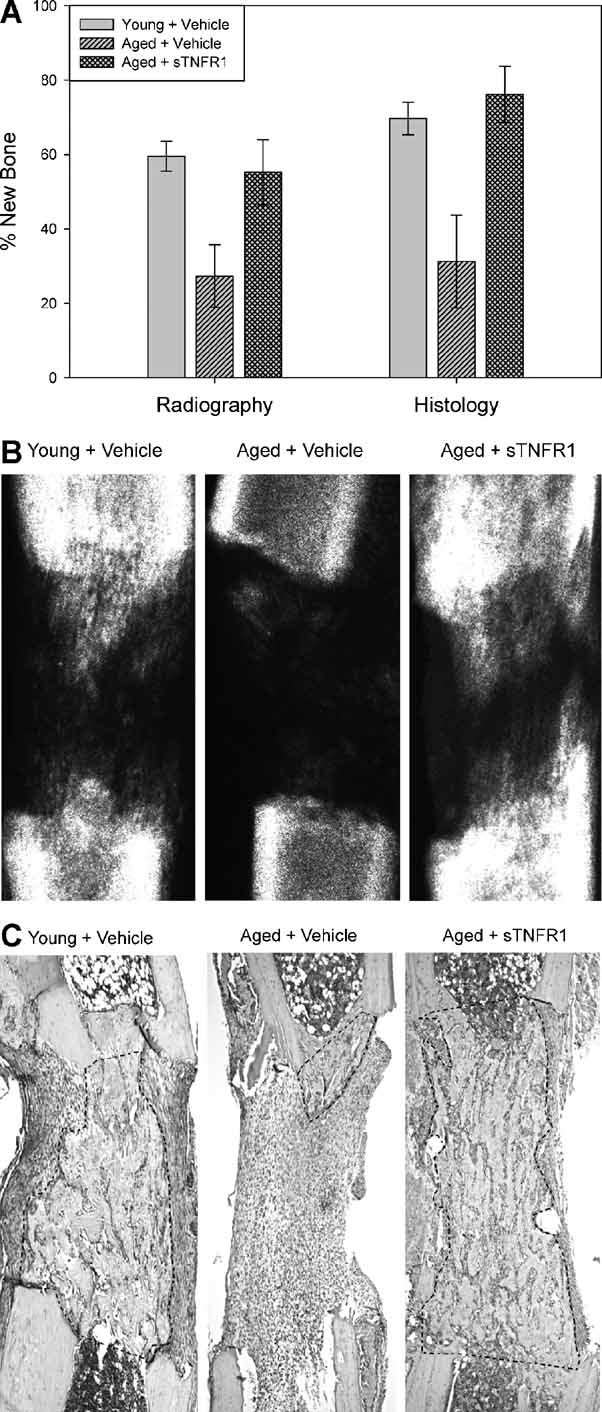
TNF blocker and aged bone repair. (*A*) Comparison of the distracted tibial radiographs and the histograms demonstrated both (1) an age-related difference in young versus old mice and (2) an sTNFR1-associated increase in aged mice in percent mineralized area and percent new bone/osteoid formation, respectively. Analysis of percent mineralized area in radiographs demonstrated the following: Young vehicle 59.5% ± 10.9% versus aged vehicle 27.4% ± 8.4%, *P* < .05, and aged vehicle versus sTNFR1-treated aged 55.2% ± 8.8%, *P* < .05. Analysis of percent new bone/osteoid formation in histologic sections confirmed the radiographs and demonstrated an age-related decrease between young vehicle (69.6% ± 4.4%) and aged vehicle (31.2% ± 12.4%, *P* < .01) and an increase in the sTNFR1-treated aged mice (76.1% ± 7.6%, *P* < .01). (*B*) Representative radiographs from young, aged, and aged sTNFR1-treated mice. (*C*) Representative histograms from young, aged, and aged sTNFR1-treated mice. The percent new bone/osteoid formation is shown outlined by dashed lines.

Radiographic analysis demonstrated a significant increase in the mineralized area of distraction gaps of sTNFR1-treated aged mice (55.2% ± 8.8%) and young mice (59.5% ± 10.9%) versus vehicle-treated aged mice (27.4% ± 8.4%, *P* < .05). Histologic analysis confirmed the dramatic increase in bone/osteoid formation observed in the radiographs of the sTNFR1-treated aged mice (76.1% ± 7.6%) versus the vehicle-treated aged controls (31.2% ± 12.4%; *P* < .01). The vehicle-treated young mice averaged 69.6% ± 4.4% (Fig. 1). In fact, 86% of the sTNFR1-treated aged mice “bridged” (osteoid from proximal and distal cortices from a continuous bridge across the gap) the gap during distraction, whereas 45% of the young and none of the vehicle-treated aged mice exhibited bridging. Serum samples were taken to compare the hormonal milieu during DO. Serum analyses confirmed high levels of sTNFR1 in treated mice only (Table 1). Further comparisons between sera from young, vehicle-treated aged, and sTNFR1-treated aged mice demonstrated (1) increased serum levels of TNF and IL-6 in aged versus young mice, (2) unchanged levels of insulin, IGF-I, IGF-BP-2 and -3 [BP2,3], and (3) decreased levels of IGF-BP-6 [BP6] and DKK-1 in both aged groups versus the young group (see Table 1). In addition, serum osteocalcin levels were measured in all groups; however, within the same experiment, only the aged controls (47 ± 2.1 ng/mL) were significantly (*p* < .001) different from the aged + sTNFR1-treated mice (69 ± 1.0 ng/mL). In comparison between groups, all the young mice had significantly higher osteocalcin levels than the aged controls.

**Table 1:** Study 1: Serum Cytokine Analysis

	TNF	IL-6	Insulin	mIGF-1	BP2	BP3	BP6	DKK-1	hsTNFR1	hsTNFR2
Aged+Vehicle	11.1 ± 1.6	21.3 ± 4.4	476.0 ± 71.0	460.7 ± 31.3	63251.8 ± 11006.4	295996.4 ± 6843.6	2526.8 ± 269.6^3^	1344.4 ± 227.8^5^	57.5 ± 19.1	4.9 ± 0.6
Aged+sTNFR1	10.9 ± 2.3	15.2 ± 6.7	557.6 ± 70.6	368.2 ± 45.5	55996.5 ± 8707.4	309917.4 ± 5053.2	1943.8 ± 261.0^4^	1770.4 ± 290.6^6^	4868.6 ± 60.7^7^	9.4 ± 5.2
Young+Vehicle	3.7 ± 0.7^1^	6.6 ± 1.1^2^	436.0 ± 91.8	366.7 ± 39.9	63034.6 ± 6892.9	303081.6 ± 6321.3	8288.7 ± 1091.4	4400.9 ± 894.5	ND	ND

Values in pg/mL except mIGF-1 in ng/mL. 1 = young vs aged, *p* < .001; 2 = young vs aged, *p* = .002; 3 = young vs aged, *p* < .001; 4 = young vs aged treated, *p* < .001; 5 = young vs aged, *p* = .02; 6 = young vs aged treated, *p* = 0.01; 7 = vehicle vs treated, *p* < .001.

In addition, to test if sTNFR1 has “anabolic” effects independent of age, we used *n* = 14 (9-month-old) middle-aged male C57BL/6 mice who received either vehicle or sTNFR1 treatment in the same protocol. Radiographic analysis demonstrated no significant differences in the mineralized area of distraction gaps between the sTNFR1-treated and vehicle-treated mice (vehicle: 68.3% ± 7.5%; treated: 73.3% ± 3.8%). This finding suggests that the “anabolic” effects of TNF blockers are age-related.

### Study 2: The effects of sTNFR1 and sTNFR2 (Etanercept/Enbrel) on direct bone formation in 21-month-old mice

**Fig. 2:**
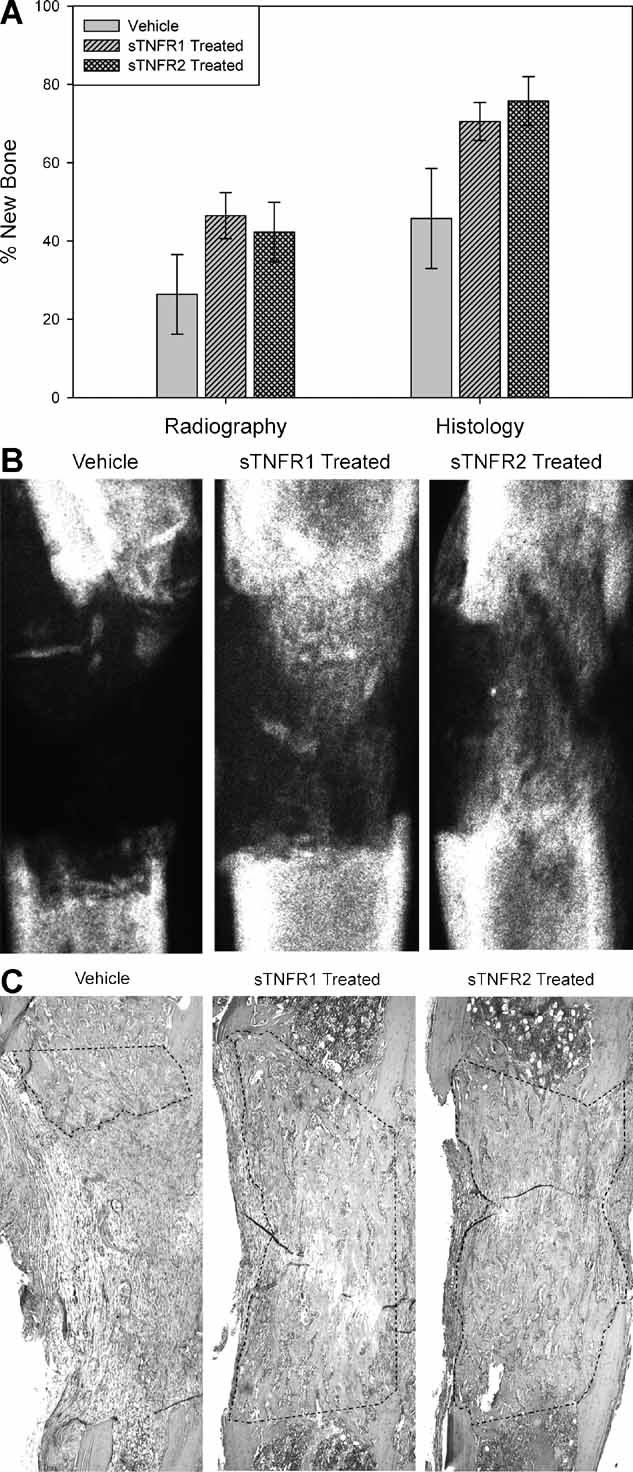
TNF blocker and aged bone repair. (*A*) Comparison of the distracted tibial radiographs and histograms demonstrated both an sTNFR1- and an sTNFR2-associated increase in aged mice in percent mineralized area and percent new bone/osteoid formation, respectively. Analysis of percent mineralized area in radiographs demonstrated the following: Aged vehicle 26.4% ± 10.1% versus sTNFR1-treated aged 46.5% ± 5.9% versus sTNFR2-treated aged mice 42.3% ± 7.6%, *P* < .05 for both treated groups. Analysis of percent new bone/osteoid formation in histologic sections confirmed the radiographs and demonstrated a TNF blocker–associated increase over aged vehicle (45.8% ± 12.8%) versus sTNFR1-treated (70.5% ± 4.9%, *P* = .03) and sTNFR2-treated (75.7% ± 6.3%, *P* = .04). (*B*) Representative radiographs from aged, aged sTNFR1-treated, and aged sTNFR2-treated mice. (C) Representative histograms from aged, aged sTNFR1-treated, and aged sTNFR2-treated mice. The percent new bone/osteoid formation is shown outlined by dashed lines.

Radiographic analysis demonstrated an increase in the mineralized DO gap area in the treated mice (sTNFR1: 46.5% ± 5.9%; sTNFR2: 42.3% ± 7.6%) versus the aged vehicle controls (26.4% ± 10.1%, *P* < .05). Histologic analysis also demonstrated an increase in new bone/osteoid formation in the treated aged mice (sTNFR1: 70.5% ± 4.9%; sTNFR2: 75.7% ± 6.3%) versus the aged vehicle controls (45.8% ± 12.8%, *P* < .05) (Fig. 2). Serum analyses confirmed the appropriate levels of sTNFR1 and sTNFR2 in treated mice only (Table 2).

**Table 2:** Study 2: Serum Cytokine Analysis

	msTNFR1	msTNFR2	hsTNFR1	hsTNFR2
Aged+Vehicle	616.2 ± 45.4	6091.5 ± 518.2^1^	3.0 ± 1.4	31.9 ± 19.8
Aged+sTNFR1	587.7 ± 33.8	5177.7 ± 576.2^2^	4638.5 ± 143.4^4^	14.7 ± 9.1
Aged+sTNFR2	606.5 ± 53.9	7423.6 ± 674.1^3^	27.1 ± 6.6	2945.1 ± 201.6^5^
Young+Vehicle	538.7 ± 21.2	4008.7 ± 119.6	ND	ND

Values in pg/mL (m = mouse); 1 = aged vs young, *p* = .004; 2 = sTNFR1 vs sTNFR2, *p* = .013; 3 = sTNFR2 vs young, *p* < .001; 4 = sTNFR1 vs aged or sTNFR2, *p* < .001; 5 = sTNFR2 vs aged or sTNFR1, *p* < .001.

### Study 3: Comparison of the effects of rmTNF on C57BL/6 versus p21 KO male mice

It has been demonstrated that (1) the DO process is highly dependent on elevated and continuous levels of cell proliferation and (2) that TNF can inhibit DNA synthesis in several cell types.(29,38,40) Therefore, to begin to characterize the mechanisms underlying the TNF, and, by extension, the age-associated inhibition of direct bone repair, we tested the hypothesis that the induction by excess TNF of p21 (Cdkn1a), a known inhibitor of cyclin-dependent kinases and therefore a putative inhibitor of DNA synthesis, would result in delayed bone formation.(41)

**Fig. 3:**
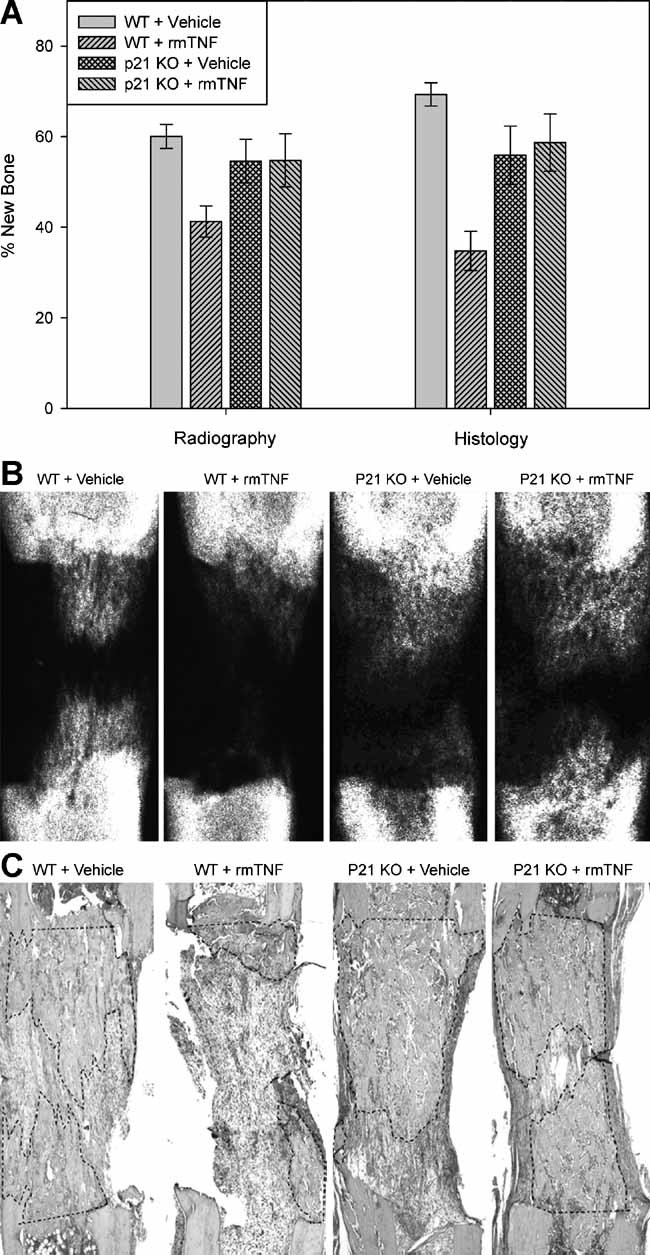
TNF and bone repair in *p21* KO mice (data values not included, just *P* values). (*A*) Comparison of the distracted tibial radiographs (percent mineralized area) and the histograms (percent new bone/osteoid formation) demonstrated an rmTNF-associated decrease in the wild type (WT/C57BL/6) in both measures (*P* < .001) but not the *p21* KO mice (*P* = .766 and *P* = .774 respectively). (*B*) Representative radiographs from young wild-type, young rmTNF-treated, *p21* KO vehicle-treated, and rmTNF-treated *p21* KO mice. (*C*) Representative histograms from young wild-type, young rmTNF-treated, *p21* KO vehicle-treated, and rmTNF-treated *p21* KO mice. The percent new bone/osteoid formation is shown outlined by dashed lines.

Radiographic analysis of the C57BL/6 mice demonstrated the expected decrease in the mineralized DO gap area in the treated mice (rmTNF: 41.3% ± 3.4%; vehicle: 60.1% ± 2.6%, *P* < .001). However, the radiographs of the *p21* KO mice demonstrated no effects of the TNF treatment (rmTNF: 55.1% ± 7.4%; vehicle: 52.2% ± 6.1%, *P* = .766). Further, histologic analyses corroborated both the expected decrease in the new bone/osteoid DO gap area in the treated mice (rmTNF: 34.7% ± 4.3%; vehicle: 69.3% ± 2.6%, *P* < .001) and the resistance to the rmTNF treatment in the *p21* KO mice (rmTNF: 58.7% ± 6.3%; vehicle: 55.9% ± 6.4%, *P* = .774) (Fig. 3). Serum samples verified that the rmTNF-treated mice had elevated levels of both TNF and IL-6, a known downstream target of TNF. TNF serum levels in rmTNF-treated C57BL/6 mice were 32.8 ± 1.1 pg/mL versus 5.4 ± 0.5 pg/mL (comparable with 3- to 9-month-old naive mice) in the vehicle-treated mice. TNF serum levels in the rmTNF-treated *p21* KO mice were 37.7 ± 4.9 pg/mL versus 12.4 ± 2.7 pg/mL in the vehicle-treated mice. Similarly, IL-6 serum levels in the rmTNF-treated C57BL/6 mice were 12.8 ± 5.7 pg/mL versus 6.6 ± 1.1 pg/mL in the vehicle controls; whereas the IL-6 levels in the rmTNF-treated *p21* KO mice were 43.9 ± 7.1 pg/mL versus 13.4 ± 4.2 pg/mL in the vehicle controls.

**Fig. 4:**
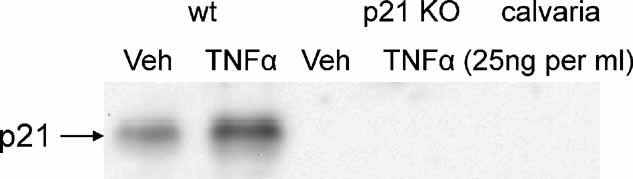
In vitro effects of rmTNF on p21 protein levels by Western blot in calvarial cultures from *p21* KO and-wild type control (WT) mice. Veh = vehicle untreated.

Based on the evidence that *p21* KO mice were protected from the osteoinhibitory effects of rmTNF, we investigated the effects of short term in vitro administration of rmTNF on calvarial cell cultures from *p21* KO and control mice. rmTNF induced p21 protein levels (Fig. 4) significantly higher than vehicle-treated wild-type calvarial cultures. As expected, p21 protein was not detectable by our antibody in calvaria isolated from *p21* KO mice, validating the specificity of the antibody. In contrast to the induction of p21 protein by rmTNF, no induction of p21 mRNA was observed in rmTNF-treated cells (data not shown).

## Discussion

TNF modulates bone formation in a dose-responsive manner; since lack of TNF signaling can inhibit bone formation, low doses can enhance osteoblast proliferation, and high doses can inhibit bone formation in vitro and in vivo.(29,30) The inhibition by rmTNF of endosteal new bone formation during DO in young mice previously reported is of a similar magnitude as that caused by chronic ethanol exposure and aging during DO in mice.(19,35) Therefore, these studies were conducted to test the hypothesis that the age-related elevations of TNF may partially mediate the deficits in bone formation/regeneration during DO previously demonstrated in these pathologies.

Here we report the results of studies on adult and aged mice undergoing DO and treatment with TNF blockers Pegsunercept [soluble human TNF receptor 1 derivative (sTNFR1)] or Etanercept/Enbrel [soluble human TNF receptor 2 derivative (sTNFR2)]. The results of these studies suggest that excess TNF acts to inhibit new bone formation in this model system and that both TNF blockers acted as potent “anabolic” agents that reversed the aged osteoinhibitory repair phenotype. We speculate that the TNF blockers are not true anabolic agents but are relaxing the TNF-specific inhibitory signal-transduction mechanisms that block existing endogenous anabolic agents. An example might be IGF-I because the serum levels actually were higher in the aged mice than the young. This could be interpreted as TNF-induced IGF-I resistance.

Several age-associated serum factors were measured. Given age-associated induction of serum TNF levels, we expected higher levels of DKK-1 in aged mice and lowered levels in TNF-blocked mice.(42) This was not the case; i.e., DKK-1 was lower in aging mice and increased in aged mice treated with anti-TNF. Several papers have demonstrated the negative effects of DKK-1 on bone in a variety of models.(42,43) However, other papers suggest that DKK-1 is necessary for bone formation.(44,45) Finally, perhaps more pertinent and in agreement with our findings, the only other paper studying the effects of age on cellular DKK-1 levels have shown decreased DKK-1 levels with age.(46) Based on the evidence, we believe that age does decrease DKK-1 levels and that the precise effects of DKK-1 on bone repair are complex and require further study. As for the IGF-BP family, members IGF-BP-1, -2, -3, -5, and -6 all have been hypothesized to be associated with age-related changes in the skeleton.(47–49) This was the justification for including several BP members in the serum analyses. No changes in IGF-BP-2 or -3 were noted, and IGF-BP-6 was lower in aging and not reversed by TNF blockers. The results do not support the preceding hypotheses, at least with regard to direct bone formation in aged mice. As for the serum osteocalcin levels, the only significant difference was noted in the aged controls (lower) versus the sTNFR1-treated aged (higher). We speculate that modulation of TNF levels has more of an effect in the aged mice than in their much younger counterparts perhaps owing to increased sensitivity to excess TNF in the aged. It may be important to note that these values are not markers of homeostatic responses to aging or exogenous TNF; rather, they reflect serum changes while the animal undergoes a significant repair/regeneration response.

We also provide evidence that TNF-induced p21 is a potential candidate for mediation of the underlying osteoinhibitory mechanisms. Evidence from the in vivo study demonstrates that *p21* KO mice are not deficient in direct bone formation during DO and that they are significantly protected from the osteoinhibitory effects of rmTNF. Evidence from the in vitro study demonstrates that rmTNF induces p21 protein in wild-type calvarial cells but not in calvarial cells from *p21* KO mice. These results are consistent with the findings that TNF induces p21 in many cell types and that p21 can inhibit proliferation in osteoblasts.(29,31,41) p21 was first described as a potent and universal inhibitor of cyclin-dependent kinases and, as such, was responsible for G_1_/S arrest after DNA damage and during quiescence. Also, p21 has been associated recently with replicative, premature, and cytokine-driven senescence.(22,31–33) Considering that (1) bone formation during DO depends on high levels of sustained proliferation and (2) that proliferation markers are significantly decreased in both age-related and exogenous rmTNF-related inhibition of bone formation, one could hypothesize that p21 is acting solely to disrupt preosteoblast and osteoblast proliferation. Consistent with this hypothesis, TNF and senescence induce cell arrest by p21 in mouse embryo fibroblasts (MEFs), which can differentiate into osteoblasts and could be considered very early osteoblast precursors.(50–52) Whether or not the DO gap contains the equivalent of MEFs remains to be demonstrated; however, we have detected markers of embryologic osteoblast precursors in the DO gap by immunohistochemistry, i.e., runx 2 and twist 2/dermo 1 (unpublished observations).(53) Further work will be needed to test these hypotheses, especially because p21 also has been shown to inhibit osteoblast differentiation, perhaps by interaction with runx 2 and/or the WNT 4 promoter.(41,54,55)

As with fracture healing, DO enhances demand for blood flow but does so to a greater extent.(14,56–59) Angiogenesis appears crucial to both direct and endochondral bone formation.(59) In fact, it has been postulated that angiogenesis occurs before osteogenesis in DO.(59) This raises questions about the effects of aging, TNF, and TNF antagonists on angiogenesis. We do measure the percent new sinusoid area in the developing DO gap, and we can identify endothelial cells by immunohistochemistry. In our experience in young, old, and rmTNF-treated mice after 14 days of distraction, direct bone formation is always accompanied by and significantly positively correlated with developing sinusoids. It has been demonstrated that chronic ethanol exposure both induced TNF and inhibited proliferation in endothelial cells.(60) Also, in endothelial cells, TNF can activate inflammatory responses and increase pathogenic angiogenesis or can inhibit proliferation and increase apoptosis.(61) Since TNF can inhibit runx 2 activity in osteoblasts, it may be important to note that runx 2 also can regulate endothelial cell proliferation.(62) Therefore, the effects of age and/or TNF on the DO gap are possibly the result of the combined effects on both angiogenesis and osteogenesis.

Common clinical analogues of the experiments proposed here are impaired fracture healing and healing after other orthopedic procedures in aged patients.(1–4) Blockage of cytokine-mediated osteoinhibitory effects also could be clinically appropriate in more orthopedic cases than just those with aging as a major risk factor. For example, TNF has been proposed to mediate orthopedic implant osteolysis.(63) These results are also consistent with recent studies that demonstrate (1) that endogenous TNF lowers maximum peak bone mass in mice and inhibits osteoblastic Smad activation, (2) that blockade of TNF increased a marker of bone formation in early postmenopausal women, and (3) that elevated inflammatory markers, including TNF, are prognostic for osteoporotic fractures.(64–66) In fact, it has been hypothesized that TNF antagonists may represent novel anabolic agents, and we believe that the data presented here represent a successful test of this hypothesis.(64) Together these results support future testing of pharmacologic interventions that modulate TNF activities for the prevention of impaired fracture or orthopedic procedure healing in aged patients.
